# Foreign Substances Simultaneously in the Trachea and Oesophagus—Report of a Remarkable Instance, with Operation and Death

**Published:** 1866-07

**Authors:** J. F. Miner


ART. III.—*Foreign Substances simultaneously in the Trachea and Oesophagus—Report of a remarkable instance, with operation and death.* By J. F. Miner, M. D.

Quite a variety of substances have been known to enter the air-passages, and such accidents are not very uncommon. The same may also be said of foreign bodies in the œsophagus; they consist commonly of bones, fragments of meat or cartilage, coin, etc., etc. Artificial teeth have been swallowed, and remarkable instances of their passing through the œsophagus are on record; sometimes, however, such substances become obstructed in their passage and produce severe and even fatal effects. When any foreign substance is lodged in the upper portion of the œsophagus a marked and deceptive symptom, may be difficulty in breathing, giving rise to the appearance of its being lodged in the trachea; suffocation might thus take place from long-continued pressure, the same as when the wind-pipe is obstructed. Generally, it will not be difficult to determine if a foreign body is in the trachea; respiratory murmurs will often point out its exact location; much, however, depends upon the character of the foreign body; smooth, unirritating substances produce less severe immediate effects, while the remote consequences may not be less serious.

If the œsophagus becomes obstructed it may be the cause of a double evil—it produces a difficulty or impossibility of deglutition, and often of respiration, acting upon both channels at the same time; cases have been reported of suffocation from lodgment of bone and other substances in the œsophagus.

The following remarkable case of obstruction of both trachea and œsophagus will be noticed with interest by all those who have had similar cases fall under their care—by all who have ever attempted removal of foreign substances by operation, from either trachea or œsophagus:

J. B., aged three years, child of Mr. B. of Lancaster, swallowed one of the new two cent coin, about April 2d, which was supposed by the family, and also by the family physician, Dr. Potter, to have passed through the canal, and to not be liable to produce any serious effects. This expectation is fully justified by repeated facts—the old one cent coin—which is much larger, having passed harmlessly the alimentary canal of such children in numerous instances. The child, however, showed signs of its lodgment, complaining of difficulty and pain in swallowing and some obstruction in respiration. Milk and fluids were taken and the child remained for two or three weeks without great change, never swallowing without difficulty and distress, which appeared increasing rather than diminishing. Three or four days previous to my visit, the difficulty in respiration had become greatly increased and paroxysms of dyspnœa became so frequent and distressing that my attendance in connection with Dr. Potter was invited, with the view of surgical relief. Upon examination it appeared certain that some foreign body was lodged in the wind-pipe; the paroxysms of dyspnœa were too severe and the constant difficulty in respiration too great, to allow of any other conclusion. Supposing that the trouble must be due to the presence of the penny, and being ignorant of any other possible causes of obstruction, it was determined to open the trachea and if possible discover and remove the foreign body. The child was slowly and cautiously brought under the influence of chloroform, and the trachea opened without difficulty or embarrassment from anything except the painful paroxysms of coughing and dyspnœa, which several times caused us to delay for some minutes in our operation. When the patient could breathe, all was well and progressing satisfactorily, but when the paroxysms of coughing and difficult respiration occurred, all hopes of success would disappear. At length when the full opening was made in the trachea, allowing free access and exit of air, one of these paroxysms commenced, and soon it was apparent that no air could be inhaled; the wind-pipe was closed to all ingress of air, and only a little could pass outward with the efforts to cough. An exploring probe was passed rapidly, hoping to remove obstruction, but nothing could be detected, and air could not be admitted to the lungs. Death came kindly to the relief of the little sufferer, and the very intelligent parents of the child allowed exploration to determine the causes of all the suffering.

A two penny coin was found imbedded in the œsophagus opposite the upper border of the sternum, the edges upon each side having caused ulceration completely through the tube, the walls of which were thickened, complete opening prevented by adhesive inflammation. This thickening of the tube caused considerable pressure upon the trachea and would of itself have proved sufficient cause of death. Added to this we found an uncooked bean which had swollen somewhat and softened by the moisture and warmth of its position; this had evidently rested near the bifurcation of the trachea, perhaps partly upon or in one of its divisions, and had thus allowed of respiration. When air was freely admitted the coughing dislodged it, and the trachea was closed—closed by a substance unlike bone or metal to be detected by a probe, but soft and yielding, and of such a shape as to close the passage as perfectly as possible. After this discovery it was remembered by the mother that the child a few days before had amused itself with some beans, and while thus playing had suffered a violent turn of coughing and strangulation, since which, she had appeared every way much more distressed.

In reviewing the main features of the case, it is readily apparent that a child with a bean in the mouth would be much more liable to inhale it into the trachea if the œsophagus was obstructed by a foreign body, itself pressing upon the wind-pipe and causing coughing and labored inspiration, than if no such obstruction existed, and such substances are by no means very infrequently drawn into the trachea in conditions of perfect health. That such a combination of maladies should be found is much more remarkable, than that it should prove impossible to overcome them and save the life of the little sufferer; indeed either one of these accidents are frequently enough the cause of death. I have a specimen I have preserved with care—a flat bone an inch in length by about one-half an inch in diameter and pointed at one extremity, which I succeeded, a few years since, in removing from the left bronchus of a young lady when almost in *articulo mortis*, and since then I never quite despair of affording relief until life has become wholly extinct. Could the exact condition of this patient have been assertained previous to operation perhaps attempt at removal would hardly have been made, and yet the urgency of such a case requires an effort, at least, for relief. I had rather the victim of such an accident die during attempts to relieve, rather than to sit inactively and make no trial. Well directed efforts will not always prove successful, but death comes more welcomly if all available means have been employed to stay its course. Dr. Potter, had, in this case, wisely delayed all operative interferenee until it was manifest relief could come from no other means. The termination was sudden, the diagnosis necessarily imperfect, but the same result was liable to occur even at the same time, without operative interference; had we known the exact condition, the same result would have been every moment expected.

The simultaneous obstruction of the œsophagus and trachea must be a rare accident; that such may have occurred is probable, but they have not come within my knowledge, and I infer since I see no mention of such condition, that it is worthy of record, partly on account of its rarity.

---

Cattle Disease in the Madras Presidency.—The cattle disease has spread to an alarming extent in Burmah, and in some cases is said to have affected the human species.

